# Critical Evaluation of the Impact of COVID‐19 Pandemic on Child Health and Wellbeing and Suggested Preparedness for Future Pandemics–A Narrative Review

**DOI:** 10.1002/hsr2.72073

**Published:** 2026-03-17

**Authors:** Ranjan K. Mohapatra, Ashish K. Sarangi, Gurudutta Pattnaik, Puneet K. Singh, Rasmita Dash, Venkataramana Kandi, L. V. Simhachalam Kutikuppala, Rudra Narayan Sahoo, Snehasish Mishra, Anita Mishra, Sourya Subhra Nasker, Sasmita Nayak, Hayam A. Alrasheed, Nawal A. Al Kaabi, Amer Alshengeti, Altaf A. Abdulkhaliq, Ali A. Rabaan, Maha F. Al‐Subaie, Saad Alhumaid, Kuldeep Dhama, Md. Kudrat‐E‐Zahan

**Affiliations:** ^1^ Department of Chemistry Government College of Engineering Keonjhar Odisha India; ^2^ Department of Chemistry, School of Applied Sciences Centurion University of Technology and Management R. Sitapur India; ^3^ School of Pharmacy and Life Sciences Centurion University of Technology and Management R. Sitapur India; ^4^ School of Biotechnology, Campus‐11 KIIT Deemed‐to‐be‐University Bhubaneswar Odisha India; ^5^ Department of Microbiology Prathima Institute of Medical Sciences Karimnagar Telangana India; ^6^ Department of General Surgery Dr NTR University of Health Sciences Vijayawada Andhra Pradesh India; ^7^ School of Pharmaceutical Sciences Siksha O Anusandhan (Deemed to be University) Bhubaneswar Odisha India; ^8^ QA Lead India RASA AI, ChatOwl Inc. 201 Spear Street, Suite 1100 San Francisco California USA; ^9^ Department of Pharmacy Practice, College of Pharmacy Princess Nourah Bint Abdulrahman University Riyadh Saudi Arabia; ^10^ College of Medicine and Health Science Khalifa University Abu Dhabi United Arab Emirates; ^11^ Sheikh Khalifa Medical City, Abu Dhabi Health Services Company (SEHA) Abu Dhabi United Arab Emirates; ^12^ Department of Pediatrics, College of Medicine Taibah University Al‐Madinah Saudi Arabia; ^13^ Department of Infection Prevention and Control, Prince Mohammad Bin Abdulaziz Hospital National Guard Health Affairs Al‐Madinah Saudi Arabia; ^14^ Department of Biochemistry, Faculty of Medicine Umm Al‐Qura University Makkah Saudi Arabia; ^15^ Molecular Diagnostic Laboratory, Johns Hopkins Aramco Healthcare Dhahran Saudi Arabia; ^16^ College of Medicine Alfaisal University Riyadh Saudi Arabia; ^17^ Department of Public Health and Nutrition The University of Haripur Haripur Pakistan; ^18^ Research Center, Dr. Sulaiman Alhabib Medical Group Riyadh Saudi Arabia; ^19^ School of Pharmacy University of Tasmania Hobart Australia; ^20^ Division of Pathology, ICAR‐Indian Veterinary Research Institute Bareilly Uttar Pradesh India; ^21^ Department of Chemistry Rajshahi University Rajshahi Bangladesh

**Keywords:** child nutrition, children, COVID‐19, COVID‐associated complication, mental health, yoga

## Abstract

**Background and Aims:**

The immediate and long‐term effects of COVID‐19 pandemic on the children go beyond just being a viral infection. A child's mental health and psychological issues like irritability, anxiety, sadness, non‐attentiveness, attention deficit and hyperactive disorder needed evaluation. Based on these study queries, an investigation was carried out to correlate the psychological state and the emergency measures in children with respect to the health and wellbeing outcomes.

**Methods:**

A web‐search across scientific databases like the Web of Science, Scopus and PubMed was undertaken for a situational review through available literature, and derive credible solutions. This narrative review included peer‐reviewed articles published between January 2020 and February 2026 in English language, focusing on children wellbeing in wake of the COVID‐19 pandemic. After analysing the authenticity and relevance of the literature, 158 articles/data were shortlisted for further analyses. The data were qualitatively validated, synthesised into discrete sections and interpreted in the context of the objective of the study.

**Result:**

Children particularly in low‐economy families were likely to experience violence and abuse if they stayed for long in high‐risk settings like refugee settlements for internally displaced community. On the contrary, the children of high‐economy families had weight gain issues owing to erratic consumption patterns of high‐calorie food (like dairy products and sugar‐rich packaged snacks) coupled with restricted or limited physical activities. Their dietary patterns and unhealthy eating habits needed monitoring and restrictions to curb obesity risk in the long run. Elevated stress, anxiety and depression levels in a child may result in cognitive impairment. The possibilities of their getting addicted, developing insomnia and acquiring non‐communicable illnesses were aspects to consider. Preventive measures like social distancing, lockdowns and shutdowns and mass vaccination drives had wider and critical long‐term impact on the young and adolescent. Formulating workable modalities to identify critical influencing factors of a pandemic and assess the impact on the children and beyond is essential for foolproof solutions to combat future occurrences.

**Conclusion:**

Identifying the associated risk factors and correlating the emergency measures and psychological health seem essential. The supply of quality food seamlessly to the affected communities and the provision of protective gears for the healthcare workers could help keep the children well‐fed and relaxed while ensuring the wellbeing of healthcare workers. Maternal and infant care, child‐health services, and life‐saving medicines and vaccines would also help ease the pressure.

**Abbreviations**
ABCD: Adolescent Brain Cognitive Development
CASP: Critical Appraisal Skills Programme
CDC: Centers for Disease Control and Prevention
COVID‐19: Coronavirus disease‐2019
CPT: cognitive processing therapy
CRPD: Convention on the Rights of Persons with Disabilities
CVT: cerebral venous thrombosis
DBIs: digital‐based interventions
DVT: deep vein thrombosis
ECHO: Environmental Influences on Child Health Outcomes
HBCD: Healthy Brain and Child Development
HIV: human immunodeficiency virus
LMICs: low‐ and middle‐income countries
MERS‐CoV: Middle East Respiratory Syndrome Coronavirus
MIRA: Maximising Investigators' Research Awards
MIS: multisystem inflammatory syndrome
MIS‐C: multisystem inflammatory syndrome in children
NIGMS: National Institute of General Medical Sciences
NIH: National Institutes of Health
OHCHR: Office of the United Nations High Commissioner for Human Rights
PRISMA: Preferred Reporting Items for Systematic reviews and Meta‐Analyses
PTSD: post‐traumatic stress disorder
RSV: respiratory syncytial virus
SARS‐CoV‐2: Severe Acute Respiratory Syndrome Coronavirus‐2
TF‐CBT: Trauma‐Focused Cognitive Behavioral Therapy
UNICEF: United Nations Children's Emergency Fund
USFDA: US Food and Drug Administration
VOC: variant of concern
VOI: variant of interest
VUM: variant under monitoring
WHO: World Health Organisation

## 1 Introduction

The fallouts of COVID‐19 pandemic on child went beyond just being an infection [1]. When the schools remained closed and the children had to stay indoors with social distancing, they experienced altered nutritional intake, unhealthy dietary habits, vulnerability to domestic abuse, anxiety and stress. It led to their unhealthy physical and mental states. They might also have had limited access to critical family and community care services, and felt socially isolated. Children more likely suffered due to altered food habit, poor nutrition, domestic abuse, worry and stress. The psychological implications of COVID‐19 on children needed to be evaluated and foolproof strategies needed to be developed to address such issues. It was critical to analyse the potential effects of long‐COVID on the children and the society for future preparedness.

A pandemic does not discriminate, including between the age groups [2], and a child's life was profoundly altered within a short duration, and seemingly ran into the risk of being among the worst victims. A child's life was impacted by sudden change in socioeconomy regardless of the age group or location, which inadvertently harmed than doing any good. The access to essential family and community care services was low owing to social isolation due to pandemic. Fearing about contracting the never‐ending COVID‐19, parents cautioned children against socialising while respecting the local and central governments' advisories. School children were deprived of numerous essential joys of life including playing in the open with peers. They lost intense pedagogy too as schools closed and the teaching‐learning platform went online [1]. A child from low‐income family was more vulnerable due to less access to internet and supporting electronic gadgets from the comforts of home. Online classes and students' evaluation systems seemed ineffective and inadequate during pandemic, and would never replace ‘contact learning’ due to which students suffered hard [1, 3]. A shift of contact learning (offline) to online was not at all smooth due to skeletal internet infrastructure and low skillsets among the numerous less‐equipped educational settings in particular and countries in general.

**Figure 1:**
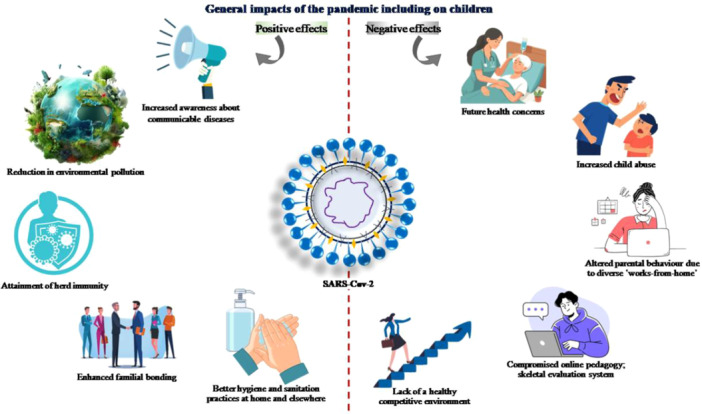
Pictographic presentation of the immediate and sustained impacts of the pandemic on child wellbeing and development [Environmental restoration, increased awareness about the communicable diseases, personal hygiene and community sanitation practices were the positives, and altered pedagogy, compromised evaluation process, parenting stress, child abuse and future health worries were the negative fallouts.].

Concerns regarding non‐health impacts of COVID‐19 pandemic on society mostly centred on the elderly and medical professionals as the most‐critical social groups. However, studies on impacts on other cohorts including children received less attention. Although less impacted by the virus directly, the children of especially vulnerable community suffered hard from indirect impacts. It included improper nutrition, compromised mental health, social isolation and extended screen‐time thereby affecting their natural vision, and limited access to healthcare and education [4]. Although children seemed to be less physically affected due to the pandemic than the adults, the pedagogy, mental health and the overall wellbeing suffered greatly. The underlying causes included the restrictions that prevented contact schooling, limited peer interactions and mobility. Increased exposure of children to the net unhealthily increased the screen‐time and exposed the young mind to abuse on virtual platform. Vulnerable children were most negatively impacted, especially the girls, that severely affected them [5]. The pandemic resulted in global upheavals and a decline in critical health determinants. Impact of COVID‐19 on particularly the children due to the constantly emerging VOC, VOI, VUM viral variants (as categorised as by the WHO) was complicated and yet unclear. The overall positive and negative impacts of the pandemic on the children are portrayed in Figure 1. Parental counseling to safe‐keep children from pandemic needed serious consideration. This narrative review discusses several factors that affect neuro‐development, academic performance, physical and mental health in children in the last 4 years since the pandemic. It aimed to decipher pandemic‐induced mental health issues in children to help the parents, health agencies, policymakers and governments to formulate mitigation measures for future.

Pandemics had short‐term and long‐term impacts on children beyond just being a viral infection. It affected mental health and psychological aspects in children as irritability, anxiety, depression, attention deficit and non‐attentiveness or hyperactive disorder. This narrative review envisaged to correlate the health and wellbeing outcomes with emergency measures and psychological state to critically evaluate the multifaceted impacts of COVID‐19 pandemic on health and wellbeing of children and suggest strategies. It attempted to ascertain the critical factors that affected child's physical and mental wellbeing, and suggested countermeasures to tackle future pandemic. The authors opine that the salient findings would not only help mitigate COVID‐19 pandemic, but the lessons learnt could also help the governments, health officials and policy makers to prepare against pandemics in the future.

## 2 Methods

### 2.1 Study Protocol

This narrative review aimed to investigate the impact of COVID‐19 pandemic on the health and wellbeing of children. The study protocol for this was prepared in accordance with the PRISMA guidelines for narrative reviews [6].

### 2.2 Study Design

The review was designed to synthesise existing literature on the manner COVID‐19 pandemic impacted children. For a broader understanding of the pandemic technicalities on child health, the trends and suggest measures for future preparedness, an in‐depth analysis of the reviews, study reports and online databases of public health agencies was carried out.

### 2.3 Literature Search Strategy

A focused web‐search across various scientific databases like PubMed, Scopus, Web of Science and Google Scholar was done to find and validate the latest literature available online. Further, the databases of health agencies like the WHO, CDC and UNICEF were also explored. In PubMed, we searched using MeSH terms and keywords, such as “stroke,” “COVID‐19,” “child,” “child health,” and “pandemic preparedness,” along with their synonyms (Table 1). Detailed search strategies for the other databases are provided in the Supplementary File (Table S1 & S2). The search was limited to articles published in the last 6 years, from January 2020 to February 2026.

**Table 1:** Search strategy used in the PubMed database.

Sl.	Keyword	Synonyms	Total hits
1.	COVID‐19	“COVID‐19” [Mesh Terms] OR “SARS‐CoV‐2” [Mesh Terms] OR “COVID‐19” [Tw] OR “SARS‐CoV‐2” [Tw] OR “COVID‐19” [All Fields] OR “SARS‐CoV‐2” [All Fields]	490,855
2.	Child	“Child*” [Mesh Terms] OR “Infant*” [Mesh Terms] OR “Adolescent*” [Mesh Terms] OR “Pediatrics” [Mesh Terms] OR “Child*” [Tw] OR “Infant*” [Tw] OR “Adolescent*” [Tw] OR “Pediatrics” [Tw] OR “Child*” [All Fields] OR “Infant*” [All Fields] OR “Adolescent*” [All Fields] OR “Pediatrics” [All Fields]	5,655,196
3.	Child health	“Child health” [Mesh Terms] OR “Mental health” [Mesh Terms] OR “Quality of life” [Mesh Terms] OR “Child health” [Tw] OR “Mental health” [Tw] OR “Wellbeing” [Tw] OR “Quality of life” [Tw] OR “Child health” [All Fields] OR “Mental health” [All Fields] OR “Wellbeing” [All Fields] OR “Quality of life” [All Fields]	1,379,787
4.	Pandemic preparedness	“Pandemic prepared*” [Mesh Terms] OR “Pandemic prepared*” [Tw] OR “Emergency prepared*” [Tw] OR “Health system resilience” [Tw] OR “Pandemic prepared*” [All Fields] OR “Emergency prepared*” [All Fields] OR “Health system resilience” [All Fields]	19,986
5.	#1 AND #2 AND 3# AND #4	(((“COVID‐19” [Mesh Terms] OR “SARS‐CoV‐2” [Mesh Terms] OR “COVID‐19” [Tw] OR “SARS‐CoV‐2” [Tw] OR “COVID‐19” [All Fields] OR “SARS‐CoV‐2” [All Fields]) AND (“Child*” [Mesh Terms] OR “Infant*” [Mesh Terms] OR “Adolescent*” [Mesh Terms] OR “Pediatrics” [Mesh Terms] OR “Child*” [Tw] OR “Infant*” [Tw] OR “Adolescent*” [Tw] OR “Pediatrics” [Tw] OR “Child*” [All Fields] OR “Infant*” [All Fields] OR “Adolescent*” [All Fields] OR “Pediatrics” [All Fields])) AND (“Child health” [Mesh Terms] OR “Mental health” [Mesh Terms] OR “Quality of life” [Mesh Terms] OR “Child health” [Tw] OR “Mental health” [Tw] OR “Wellbeing” [Tw] OR “Quality of life” [Tw] OR “Child health” [All Fields] OR “Mental health” [All Fields] OR “Wellbeing” [All Fields] OR “Quality of life” [All Fields])) AND (“Pandemic prepared*” [Mesh Terms] OR “Pandemic prepared*” [Tw] OR “Emergency prepared*” [Tw] OR “Health system resilience” [Tw] OR “Pandemic prepared*” [All Fields] OR “Emergency prepared*” [All Fields] OR “Health system resilience” [All Fields])	172

### 2.4 The Inclusion and Exclusion Criteria

#### 2.4.1 The Inclusion Criteria

The inclusion criteria under the study were as follows:

- The literature only in English (and no other) language were considered.
- The search was limited to articles published between January 2020 and February 2026.
- Only the articles published in well‐known peer‐reviewed journals were selected.
- All article types including commentary, review, correspondence, research paper, case report, concept paper, technical report and datasets were included.
- Most pertinent and recent literature based on precise search strings, as per selection criteria cited above, were included.

#### 2.4.2 Exclusion Criteria

The exclusion criteria were:

- Any duplicity of articles was excluded.
- Non‐peer‐reviewed article, like preprints and conference proceedings, was excluded.
- Articles not linked to children, even remotely, were excluded.
- Articles with apparent unreliable or inconsistent findings were excluded.

### 2.5 Data Extraction

Following the inclusion and exclusion criteria, a total of 158 articles were shortlisted for in‐depth analyses. The designated team extracted key information and data from these articles that included authorship, publication year, journal, study design, abstract, results and key findings. The extracted data were verified by two designated authors and validated independently by the third to resolve disagreements or discrepancies. The references were validated and listed manually.

### 2.6 Data Synthesis and Result Interpretation

The data were synthesised qualitatively into distinct sections to facilitate organised findings, covering mainly the impact of the pandemic on children, immunisation schedule and effect, other socioeconomic factors and the coordinated strategies by health agencies for child health and wellbeing. The findings were interpreted in light of the existing literature available online. The process followed the PRISMA guidelines (for narrative reviews), to ensure clarity and transparency.

### 2.7 Quality Assessment

Critical Appraisal Skills Programme (CASP) tool consisting of 10 questions was used to assess the quality in evaluating the study aspects [7, 8]. A detailed summary of evidence strength by topic area is tabulated in Table S3.

## 3 Results and Discussion

### 3.1 Effect of Lockdown

The overall effect of lockdown on the mental and physical health of children, understood better with the reopening of schools after the pandemic, showed the initial signs of recession. Schools reopened that complied with the numerous preventive measures to counter COVID‐19 spread, and few were hospitalised after contracting the infection. As majority of the data was derived from surveys from the real‐world scenario, the effects of these metrics on aspects like padagogy, accessible health infrastructure and physical or mental health were relatively little known [9]. Before children and adolescents could receive high‐quality, culturally‐appropriate and rights‐based mental healthcare, there were additional global issues to solve. Predicting short‐ and long‐term effects of pandemic on mental health of child and adolescent was challenging. Directing developmental and longitudinal research and implementing evidence‐based action plan for pandemic was important to manage mental and social wellbeing of children and adolescents [10].

During shutdown in China, there was decrement than increment on indicators like emotional neglect and abuse, physical neglect and abuse, sexual abuse and witnessing domestic violence in children and teenagers (ranging between 5.1% and 9.1%). Sexual abuse incidents considerably rose to 2.9% from 1.6% 8 months after the lockdown was lifted. Sadness, anhedonia state and psychotic symptoms were gender‐linked and at baseline to higher sexual abuse rates after lockdown [11]. Compared to before the pandemic and during pandemic, the data related to issues like depression symptoms significantly raised as pandemic receded. Lockdown was associated to reports of child maltreatment, though the findings contextually varied. School closures and social isolation likely contributed to increased exposure to domestic violence and depleted mental health (anxiety and depression) [12].

The attitude and behaviour of the 1–5 years old young and relatively older (6–10 years) children, as also their sleep patterns, familial relationships, digital gadget usage, educational activities, distance‐mode learning, and mood swings (like anger, tantrums and irritation) were assessed [13]. The study included other factors like child age, gender and number of siblings. The findings showed no discernible variations in the quality of sleep (73% in 2020% and 72% in 2021) between preschool‐aged, while the sleep was substantially impact in older children with 34% sleep disturbance in 2020% and 27% in 2021. Additionally, 52% of the older children experienced attention shifts during the initial lockdown and the number rose to 59% in 2021, indicating increased disturbed attention [13]. The study found persistence and a rise in irritability, anger and tantrums in both preschoolers and older children.

A study in India examined the impact of lockdown on sleep and psychosomatic issues in children during lockdown, and correlation between these factors and screen usage [14], enrolling the children of 1–12 years from a tertiary care hospital in south India. It was found that the majority of children under five had less than 2 h daily screen time, and 58.16% of children of 5–12 years had more than 4 h daily screen time. It concluded that while a large percent of participants between 5 and 12 years experienced visual issues, children younger than 5 years exhibited significant behavioural abnormalities and sleep issues.

The emotional and behavioural disruptions in children after lockdown were assessed in an earlier study, in children between 6 and 12 years of age admitted as outpatient (in OPD) in a tertiary care hospital. It was found that those who were confined or had limited mobility were more likely to have extended screen time, had sleep issues, or exhibited aggressive behaviour [15]. There issues were more externalising in males and internalising in females. Normal and mentally healthy children experienced neurobehavioral abnormalities owing to lockdown.

The long‐term monitoring and the provision of mental healthcare services especially among the young were extremely important for public health [16]. Based on diversely defined overall recovery from persisting symptoms, a total of 22 studies proposed variable estimates ranging between 0% and 67% in 8–12 weeks and 8% and 51% in 6–12 months. Data on using rehabilitation resources were scarce. The burden of persisting symptoms was more in the infected than in the control (negative) or influenza cases although the mental quality between an infected and a non‐infected child did not differ significantly, according to controlled trials.

### 3.2 Food Habits

A recent Polish study on influence of lockdown on dietary habits, sleep and social media usage on children and adolescents found altered dietary habit and daily activity patterns [17]. Closure of marketplace, schools, corporate houses, entertainment places and other social gathering places affected social activities. A Saudi Arabia, Britain and Turkey study found weight gain in children due to high consumption of nutritious food and staying home without physical activities during lockdown [18]. Children equally chose to consume high‐calorie home‐cooked and packaged food without discrimination [19]. Gender‐specific child nutrition differed significantly, being better in boys than in girls. Thus, the pandemic affected child nutrition and weight gain significantly that might have affected healthy wellbeing in children, that might affect them in future.

Nutritious diet ensured stable organic growth and development of immunity‐protected child [20]. Even though breast milk provided the needed nutrition up to 6 months and protected, it was insufficient after 6 months. So, various foods like fruits, vegetables, grains, pulses, nuts, rice, animal and dairy products were needed alongside. Having a small stomach, a child needs to eat healthy snacks and quality potable water frequently between meals. Over‐the‐counter packaged food in the marketplace like soft drinks, sweets and savoury snacks with high salt, sugar, fat and preservative chemicals could hamper healthy and organic growth [21]. Further, formula milk or formulated baby‐food may be unhealthy for children as they contain unhealthy fat levels, and high sugar and salt. Parents either could not afford quality food or move out to buy those due to disrupted life during pandemic. The pandemic profusely altered conventional lifestyle, health, socialisation, and so forth. in the ‘new normal’ and ‘stay‐at‐home’ increased the screen time [22] which was associated with weight gain in children. Maternal malnutrition from preconception to post‐partum stages could also be associated to malnutrition in particularly the breast‐fed child. This led to difficult labour and affected both mother and the child in long‐run [23]. Hasty measures to counter and mitigate pandemic resulted in reduced food security, healthcare and education [23]. It resulted in low birth weight in the newborn, post‐partum difficulty, chronic undernourishment in child and even a higher rate of morbidity and mortality. Even the avoidable paediatric ailments seemingly could worsen later due to interrupted immunisation drives during pandemic. An action‐plan was essential to enhance the general health in post‐pandemic era paying attention to the vulnerable population [23].

As mentioned earlier, the true impact of the pandemic on children was well beyond an infection. It had public health implications with life‐long consequences [24]. Targeted effective measures mainly for vulnerable households and children were needed to guarantee a child's basic rights of nutrition, education, health and development. Eating habits had significant short‐ and long‐term health implications in children and adolescents, critical for physical growth and mental health [21]. Fast‐food, junk‐food and soft drinks intake allegedly reduced in children [25]. An Italian cross‐sectional online survey showed altered increased consumption of sweet packaged snacks (34%), processed meat (25%), and pizza, bread and bakery products (47%) [26]. Increased consumption of vegetables, fresh fruits and legumes (19%) was also observed. Body weight gain in children was associated with high consumption of sweet packaged snacks and dairy products, while the same was linked to increased intake of comfort foods and processed meat in adults [27]. Thus, there was a need to monitor unhealthy eating patterns using tele‐health to manage and prevent pediatric obesity during pandemic, deliver nutrition and targeted workouts [28].

### 3.3 Impacts on Children in the Low‐ and Middle‐Income Countries (LMICs)

As per secondary data preliminary survey from literature, eating habit and lifestyle of child were significantly disrupted. While nutrition inadequacy was predicted to worsen in LMICs, obesity was predicted particularly among the vulnerable groups in the developed and developing world. It is bound to progressively extend social inequalities, and health and wellbeing gaps. Pandemic impact on the young and adolescents was intangible, much beyond the viral illness that was tangible. Community health effects of the pandemic may have long‐term adverse effects on children, more at the mental level than physical. For nutrition, health and proper organic growth, effective and focused interventions at the best possible level at households were needed, particularly among vulnerable young population, to ensure fundamental child rights [5].

Concerns about the risk of the pandemic in the young with high comorbidities, impact on health, economy, and the ability of prevailing health systems to handle additional burden of pandemic grew as the pandemic spread to LMICs. Children in the LMICs make up a sizable population and might be at a high risk for severe malnutrition and lower respiratory infections [29]. Despite it, a direct effect of COVID‐19 was less concerning in children, who remained largely asymptomatic or developed mild illness as was witnessed in the high income (developed) countries. Child care might be further impaired as such resources were diverted to counter epidemics among adults. SARS‐CoV‐2 transmission in LMICs was attributed to compromised living conditions with low hygiene and sanitation, logged water and public crowding. Inadvertent unseen consequences of the pandemic on child's health are extremely concerning. The factors contributing to it were poverty, interrupted education, inaccessibility to the nutrition programmes at schools, decreased access to medical facilities, and disrupted immunisation and other child healthcare initiatives [29]. Analyses revealed three main concerns: 1. balancing home‐schooling with the work‐from‐home activities of parents; 2. improving parents' relationships with schools, health support services and agencies; and, 3. understanding the effects of emotional and physical health of homeschooling kids and parents [30].

Maharashtra, India implemented a comprehensive rural community‐based intervention COVID‐Free Village Programme (CFVP) to assess COVID‐19 control and resilience measures, involving rural Pune and Stara residents as participants [31]. Pune exhibited a noticeably high combined COVID‐19 awareness index (0.43; 95% CI: 0.29–0.58) compared to Satara. Pune complied better with COVID‐appropriate practices like masking (17%; 95% CI: 0–38) and hand‐washing (23%; 95% CI: 3%–45%) than Satara, illustrating how awareness impacted behavioural patterns across geography within comparable demography. Assessing how hand sanitisation recommendation impacted diarrhoea during COVID‐19 using national and sub‐national data from District Health Information Management System (DHIMS 2), a study compared diarrhoea prevalence between pre‐COVID and COVID‐19 eras in Ghana [32]. It was found that, the average diarrhoea cases reduced significantly (*p* < 0.001) during the pandemic as opposed to earlier that could be attributed to improved hand sanitisation.

A 3‐year longitudinal study in India evaluated the efficacy of trainer workshops for teachers and students involving innovative educational tools on hand‐washing and hygiene practices, wherein above 200 schools and above 5000 pupils participated [33]. The concluded that 92.28% instructors implemented the learning in classrooms, and that 58.16% kids had a better awareness of germs and the benefits of hand‐washing after the workshop. Further, all the teachers reported a reduction in childhood diarrhoea and vomiting connected to poor hygiene. In another study, the cleanliness and safety measures were evaluated 311 parents and kids from Barika refugee camp in Kurdistan, Iraq during the COVID‐19, and compared with pre‐COVID scenario. It was found that 61.09% of them washed their hands frequently and 58.58% did it frequently during COVID‐19 than they did it before [34]. It was also discovered that kid protection, disinfectant types and water consumption priorities altered dramatically during the pandemic, and the determinants of these changes were primarily the age and educational level of the women folks.

### 3.4 Impact of Online Education on Children

Due to the imposed online ‘over‐time’, spending extended hours on the internet, a child was more susceptible to unsolicited contents and online predation [35]. Sexual misappropriation and cyber‐bullying issues as side‐effects worsened due to extended internet usage and unsupervised online engagement [36, 37], exposing the children to mental health and wellbeing challenges. House‐confined, domestically displaced or refugee children are particularly susceptible. Children of particularly the internally displaced population in violence and refugee settlement areas were more vulnerable. They likely witnessed or experienced violence and abuse during lockdowns, shutdowns and in‐place‐shelter norms. Without an alternate source, 368.5 million children in 143 nations relied on school meals often as a good daily nutrition source, because of which it was anticipated that online classes could have contributed to increased malnutrition.

Elevated inflammation markers levels like erythrocyte sedimentation rate and the C‐reactive proteins was crucial to assess COVID‐19 impact on children, and help design strategies to influence at individual, society and national levels. Detecting infection early in vulnerable children was vital to invoke appropriate and timely medical care. The severity in COVID‐19 affected children was often linked to their underlying health status or cardiac involvement. Initial cardiac signs typically manifest with cardiovascular changes. These include reduced left ventricular systolic function with less than 60% ejection fraction, diastolic dysfunction, arrhythmias, ST segment changes, prolonged QTc and premature atrial or ventricular beats. Assessing ventricular function and measuring cardiac enzyme levels could serve as reliable indicators to predict severe clinical manifestations in children. It also significantly delayed SARS‐CoV‐2 variants' evolution, as studies suggested.

### 3.5 Immunisation in Children

Lower vaccination coverage in children than adults (due to underlying technical and social reasons) was a possible barrier in curtailing the spread of pandemic. While late availability of vaccines for the young was the technical reason, the fear/worry of parents about vaccine safety was the social reason [38]. In addition to rising risk of developing severe MIS (MIS‐C), SARS‐CoV‐2 impacted scholastic performance and psychology in children that are critical in upkeeping and advancing social order. Clinical signs of SARS‐CoV‐2 infection, vaccine inefficacy, vaccination‐associated adverse events alongside a child's distinct immune systems were reported [38]. Due to relatively low instances and risk of COVID‐19 in children compared to adults and the uncertainty surrounding the relative effects of the disease and vaccine, the risk:benefit of child immunisation was more complex [39]. Parental preparedness to vaccinate children was moderate, influenced by numerous factors. The major determinants in parents' intent to vaccinate were family income, perceived COVID‐19 threat, and attitude to vaccinate (vaccination history of parent and child against influenza, confidence on vaccines, and COVID‐19 vaccination rate in parents) [40]. Policymakers need to alter misconceptions and ensure widespread (community‐level) COVID‐19 vaccination by deciphering the reasons behind vaccinate hesitancy [40]. Given the wider advantages child vaccination provided, it makes good sense to conduct COVID‐19 vaccination scientifically, uphold the moral and ethical standards, and minimise financial burden on families [41].

After the WHO declared COVID‐19 as pandemic, lockdowns and shutdowns were implemented to restrict public movements [42, 43, 44]. Initially, the children were the least exposed; they were restricted to home, studied online, and were under strict parental care and supervision. Thus, the disease course and potential COVID‐19 consequences in children were ill understood. COVID was construed to be asymptomatic, mild and self‐limiting in children with gastrointestinal symptoms to be more profound compared to respiratory symptoms [45, 46]. However, children suffered severe infections that required hospitalisation including ICU admission. Children had better disease outcome in severe COVID‐19 as compared to the adults [47]. Child vaccination against COVID‐19 was increasingly relevant nonetheless.

Attributed to less‐severe infection in children that rarely required hospitalisation, vaccinating a child below 12 years had uncertainties on the need and consequences. However, vaccinating children is increasingly being considered by health administrations due to constantly emerging variants and to ensure normalcy wherein children could go to school and socialise [48]. It was more due to their role in community‐level viral transmission [49]; children were assumed to be the major infection source in the elderly. Child vaccination could, it was estimated, reduce community infection rates that could otherwise increase morbidity and mortality in such outbreaks in future as the induced individual‐level immunity protection waned [50]. It might also lead to seasonal epidemics and outbreaks‐like situation.

There are opinions against child vaccination based on available data, suggesting that COVID‐19 related morbidity and deaths were directly correlated with the age of patient [51]. Disease severity and mortality rates increased with increasing age wherein the mortality rate in children remained extremely low ( ~ 0.0016) compared with the elderly ( ~ 5.6%). The infection increased by 20% among children of 14‐17 years in fresh COVID‐19 cases, as the adults were extensively vaccinated while the children were not [52]. Studies revealed the factors that favoured those that were against and the uncertainties of vaccination. Mild infection, vaccination hesitancy and ethical concerns were the factors against vaccinating children. Similarly increasing infection among children, easing offline study, helping to socialise and protecting the community from infection transmission were the factors that favoured the cause.

As evidence of critical impact on the young mount, beneficence, nomaleficence, autonomy and justice, morality of COVID‐19 appropriate measures like school closure and restricted public movements (in lockdown and shutdown) were questioned [53]. Although countries started vaccinating children against COVID‐19, the scenario in other countries that decided late was particularly hard with uncontrolled community transmission. This was more glaring in LMICs due to the lack of resources for rigorous implementation [54, 55].

Although vaccinating the children did not prevent SARS‐CoV‐2 transmission, it could minimise the severity like the debilitating and potentially life‐threatening MIS in children (MIS‐C). Some 5–11 year old vaccinated developed MIS‐C after weeks of infection [56]. Some reported side‐effects like acute coronary syndrome, cardiac arrest, CVT, DVT, hypertension, myocardial infarction (MI), MI with nonobstructive coronary arteries (MINOCA), myocarditis, pericarditis, pulmonary embolism (PE), stress cardiomyopathy, tachycardia, vaccine‐induced thrombotic thrombocytopenia (VITT) and other venous thrombotic disorders [57]. Of these, myocarditis and thrombosis (particularly VITT) were more frequently reported. Political interference and other non‐health‐related variables could greatly impact vaccination consent by parents. Health agencies, researchers and clinicians could significantly address it through trust, clarifying vaccination myths and instilling confidence [58]. Addressing vaccine‐related concerns by healthcare providers with compassion and empathy, emphasising that the best interests of the child should always come first, could serve as the foundation for it.

### 3.6 COVID‐19 Vaccines for Children

COVID‐19 vaccines for children are four, inactivated, subunit, RNA/DNA and viral vector based [59]. Among these, mRNA/DNA and viral vector based vaccines induced adequate neutralising antibodies and efficient T‐cell immunity. Although subunit vaccines induced better neutralising antibodies, T‐cell stimulation response was reportedly weak. Reports confirmed that children were not included in clinical trial of RNA/DNA/protein‐based and subunit/inactivated/viral vector‐based vaccines, majorly due to ethical considerations to involve children as subjects in trials. USFDA approved the Pfizer/BioNTech and Moderna vaccines to vaccinate 16–18 year adolescents [60]. Vaccinated children exhibited better neutralising antibody titre and efficient T‐cell immune response, despite sustained immunological response including T‐cell immunity and neutralising antibodies at par with the adult population after SARS‐CoV‐2 infection [61].

US‐CDC recommended Spikevax of Moderna, US and COMIRNATY of Pfizer‐BioNTech, Germany as the two COVID‐19 mRNA vaccines for children and adolescents. Approved for ‘emergency use’ after demonstrating high efficacy during the clinical trials, these were made available for children older than 6 months. The CDC stated that a vaccination decision was carefully made by the physician after the consent of the parents to voluntarily participate. While two Moderna vaccine Spikevax doses with 4–8 weeks gap was recommended for children of 6 months to 4 years, vaccination schedule for COMIRNATY of Pfizer‐BioNTech was in three doses at 0, 3–8 weeks, and at least 8 weeks after the second dose [62]. Novavax (by Serum Institute of India) could be administered to children over 12 years. The United Kingdom suggested vaccinating children below 15 years [63]. BNT162b2 mRNA vaccine of Pfizer–BioNTech was approved in the United Kingdom to immunise children of 5–15 age group [64]. However, its efficacy was moderate to low as Omicron variants circulated in Italy (29.4%; 95% CI: 28.5–30.2) and the US (65%; 95% CI: 62–68) [65, 66]. It demonstrated that the immunity waned 2 months after vaccination, showing that COVID‐19 vaccine had limited efficacy to protect sustainably, stressing on the need of more effective vaccine and booster doses.

A survey‐based Indian study involving 97.3% pediatricians from 23 states revealed that around 60% respondents knew about children vaccines Covaxin and Corbevax [67], and about 50% were unsure about the safety, efficacy and vaccination scheduling. It was found that about 60% pediatricians felt that school‐level vaccination drive might improve vaccination coverage among children, and vaccinating the children even with comorbidities was also important. About 75% of the 533 children in a study were administered Pfizer‐BioNTech vaccine, 23% by AstraZeneca and 1% by Moderna. It was found that 28.3% vaccinated had severe neurodisabilities, followed by congenital disorders (9.4%), learning disabilities (9%), chronic respiratory diseases (9.6%) and Down's syndrome (5.4%). Fever, headache, fatigue and flu‐like symptoms were the commonly observed symptoms after vaccination, the latter being the most common after first and second doses. Only 2% of the vaccinated had aggravated adverse effects, and needed hospitalisation.

### 3.7 COVID‐19 Complications

The perception that children are likely to be less affected by COVID‐19 pandemic is only partially true as they invariably stayed indoors during pandemic with no clue to validate the perception. Impact and complications of COVID‐19 in children were frequently reported. Multi‐systemic inflammatory syndrome among the pediatrics led to significant morbidity and mortality due to severe inflammation that damaged the heart, kidneys and other internal organs [68, 69]. Physical and psychosocial ill‐effects in children during lockdown increased; the impact was evident in children who already had chronic illnesses like rheumatic disease, multiple sclerosis and neurodevelopmental issues [70, 71, 72, 73]. Haemoglobinopathy children were only moderately affected during the pandemic unlike during the H1N1 pandemic earlier when they were severely affected. Children potentially at a risk of SARS‐CoV‐2 exposure might develop hepatitis as evidenced through the rising hepatitis incidents as the COVID‐19 restrictions were eased [74].

Side‐effects of Moderna's mRNA‐1273 and Pfizer‐BioNTech's BNT162b2 immunisation in children below 2 years were both systemic (38.8%) and local (21.1%) after initial dose [75]. Fever (13.8%), injection‐site reaction (21.1%) and irritability or fussiness (30.1%) was seen. 18 (0.7%) of the 2633 children reported critical reactions, and 87 (1.5%) of the 5644 participants needed medical attention (BNT162b2: 1.8%; mRNA‐1273; 1.4%). With no recorded fatality, seizure or febrile seizure was observed in six. Vaccinated and unvaccinated Canadian children and adolescents ranging from 6 months to 19 years were enrolled in a cohort study [76]. 131,032 participants received two doses, 259,361 received single dose, and 1179 were control. Vaccinated adolescents experienced greater health events in the week after the second dose than unvaccinated adolescents (4.6% who received BNT162b2, 8.5% who received mRNA‐1273 and 10.6% who received heterologous vaccine, as compared to 3.7% in control). The majority of these were self‐limiting and resolved in a week.

Myocarditis and pericarditis incidents vary in pediatrics. While younger age‐group did not report health issues as frequently as unvaccinated ones, adolescents who received second mRNA COVID‐19 vaccine dose did. The cases in male adolescents with myocarditis and/or pericarditis peaked in 0–28 days after second dose, in three (0.037%) of the 8088 homologous BNT162b2 recipients and two (0.529%) of 378 homologous mRNA‐1273 recipients. An earlier systematic review investigated the prevalence, clinical manifestation and correlation between COVID‐19 vaccination and myocarditis and pericarditis in children and adolescents [77]. Of the 33 patients, BNT162b2 was administered to 32 (96.9%) and mRNA‐1273 was administered to one (3.03%). Myalgia (*n* = 15, 45.4%), headache (*n* = 9, 27.2%), fever (*n* = 18, 54.5%) and chest discomfort (*n* = 31, 93.9%) were witnessed. As per clinical examinations, there was an increase in ST (*n* = 32, 97%), CRP (*n* = 9, 27.2%) and cardiac troponin (*n* = 29, 87.8%). Myocarditis and pericarditis were more common in male children and teenagers, and frequently reported in BNT162b2 recipients.

Long COVID refers to SARS‐CoV‐2 infection symptoms in children that would last for 3 months or longer. While kids would cure from COVID‐19, some developed fresh symptoms and others had ongoing issues. Long COVID impacted children with minimal COVID‐19 symptoms. As much as 20% children having a history of SARS‐CoV‐2 infection might manifest long COVID. Nearly six million children in the US might be affected by this, which was more than the number of children who had asthma, a common chronic ailment in children [78]. Research on pathophysiology of chronic COVID in children was ongoing, with possible autoimmune, neuroinflammation and viral persistence factors. Although the exact risk factors for long COVID in children are unknown yet, research indicated that children experiencing acute SARS‐CoV‐2 infection or those with comorbidities were more vulnerable. Long COVID in children might present respiratory symptoms like coughing and dyspnea, and general and neuropsychiatric disorders like headaches, muscle weakness and exhaustion [79].

Prolonged symptoms were more prevalent among the young children who had infection history than in those without, as per Pediatrics cohort study Researching COVID to Enhance Recovery (RECOVER). It comprised of 539 preschool‐age children and 472 infants/toddlers. Children with SARS‐CoV‐2 infection history were assessed for poor appetite, sleeping issues, wet and dry cough, and stuffy nose (babies and toddlers), and for daily fatigue, drowsiness, low energy and dry cough among preschool‐age children. According to findings, 40 of 278 (24%) and 61 of 399 (15%) of infected infants and toddlers were susceptible to long COVID [80].

### 3.8 Skeletal Medical Support to Children

Studies investigated the effect of child maltreatment that prevailed during the pandemic, and compared it to the pre‐pandemic time. Target population and different types and measures of child maltreatment, the studies seemed controversial and less acceptable owing to source data. A few systematic reviews from the collected literature studied the prevailing and altered healthcare services in children during the pandemic [81]. Financially weak (particularly with single earning member) families could not afford a decent education attributable to the prevailing global economic slump. Thus, it led to more fatality in children, undoing the gains of reduced mortality in the newborns in previous years. The numbers were scary and reflected a critical relationship between economy and mortality as impact of the crisis, which was seemingly underestimated. It also did not account for disrupted services due to the pandemic.

Human life, including the school‐going children, thrived in the virtual space during pandemic. Children and teenagers particularly suffered due to abrupt switching to virtual (online) learning and remaining detached from physical activities and social interactions. Their frequent indulge in social media and mobile games worried the parents, paediatricians and psychologists equally. Data suggested that the internal and external behaviours were grossly affected in the pandemic‐impacted children. A multidisciplinary approach and thorough assessment could help address such concerns [82]. The majority of the vulnerable children were from low‐income community living in small houses and parents were also poorly educated, and this socioeconomic disparity affected mental health. Home‐confinement and social distancing during pandemic were the critical risk factors directly or indirectly linked to mental health status. Other unhealthy lifestyle factors (like extended screen‐time, less physical activity and disturbed sleep) during pandemic increasingly stressed the parents and affected their mental health too [83].

### 3.9 Impacts on Child Health and Wellbeing

Children and adolescents across age‐groups suffered critically during pandemic, across globe. Pandemic‐imposed restrictions profoundly affected the health and wellbeing that could be for a lifetime for some; pandemic widely disrupted education ever in the annals of history, affecting about 1.6 billion in over 190 countries. The August 2020 WHO Pulse survey on essential health services noted that 90% of the countries reported disruptions since the onset of pandemic. These included routine immunisation of children, outreach (70%) and facility‐based (61%) services. Harmful fallouts of the pandemic were unequal, and children from vulnerable communities were disproportionately affected, with long‐term health consequences [84].

With multiple SARS‐CoV‐2 variants continuously emerging, understanding whether symptoms and impacts of novel variants varied in children was essential. Morbidity and mortality in child reportedly increased with bacterial, fungal and respiratory viral coinfections. Though coinfection risk of COVID‐19 infected children was lower than adults, clinical outcome in children was severe, associated with RSV and mycoplasma pneumonia coinfections [85]. Effective diagnostic and therapeutic measures particularly in children were thus suggested. Severe acute hepatitis, vomiting, jaundice, diarrhoea, abdominal pain and malaise during pandemic with unknown aetiology in seemingly healthy children are reported [86]. Acute hepatitis with high liver enzyme levels was manifested, and the WHO had reported 1010 suspected cases by July 12, 2022 [87]. Indian states reported hand, foot and mouth disease (HFMD, tomato fever or flu with blisters) in children in April and May 2022 [88], symptoms close to mpox reported in many counties.

The imposed restrictions during pandemic coupled with the deadly influenza and Ebola viral infection among others reportedly affected childcare services. Children suffered from high stress, anxiety and depression that could have led to cognitive impairment [89, 90]. Their suffering from insomnia, drug abuse and developing life‐style diseases were suspected. A multi‐location study involving 51 centres and hospitalised children showed high hospitalisation. Hospitalisation was high in Brazil (11,613 hospitalised, 10% invasive ventilation, 7.6% mortality) and Japan (1038 hospitalised, 2.1% on respiratory support, 0 ventilation or mortality). A study in India concluded that referral bias of hospitalisation was a factor behind increased reports. The features of the 65 children cohort including mortality and critical disease risk factors were covered in detail and the findings were of greater significance [91]. Long COVID (post‐COVID syndrome) were the terms coined to describe long‐term COVID‐19 symptoms [92]. Uncertainties about the risk of long‐term clinical consequences of SARS‐CoV‐2 infection in children prevailed. Multisystem inflammatory syndrome in children (MIS‐C) that emerged soon after the pandemic was potentially fatal [93]. Due to major inconsistencies among studies involving children, it was challenging to determine the precise incidence of long COVID. The risk variables to determine whether recovery occurred and if yes then how soon it was in adults than in children differed.

A high degree of variability (1.6%–70%) in symptoms prevalence was found in studies reporting long COVID among pediatric population. Headache (3.5%–80%), chest tightness or pain (1.4%–51%), fatigue (2%–87%), arthralgia (5.4%–66%) and dyspnea (2%–57.1%) were common. Findings included the limitations in daily activities, altered brain image and transient electrocardiographic abnormalities [21]. Quality of life in adolescents was unaffected by COVID‐19 as considerably as in the young and those with severe comorbidities. Although results were inconsistent, there was a decrease in daily activities and increased school absentees in COVID‐19. Available data on rehabilitation and resource needs were low. Data on 38,152 children and 6630 adolescents with long COVID were compared to a control group of 147,212 children and 21,640 adolescents with negative or no testing in a controlled study. The majority of them had mild or asymptomatic SARS‐CoV‐2 infection 4–9 months earlier. The study showed that the ‘positive’ individuals had higher symptoms rates (61.9% vs. 57%, OR: 1.22 95% CI: 1.15–1.30) compared to control, and high percentage of school bunking (above 2 weeks) [94]. A follow‐up after 6‐month of pediatric MIS observed in 38 children found that 24 (65%) had no impairment, 10 (27%) had mild impairment and 3 (8%) had significantly impaired quality of life [95].

**Figure 2:**
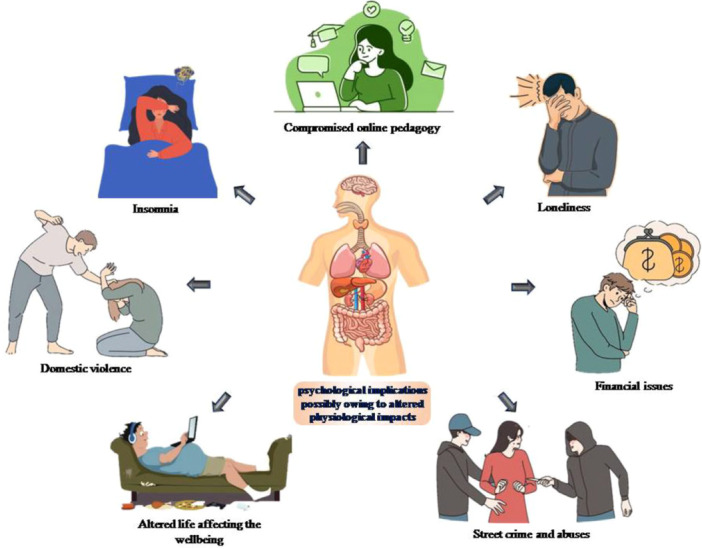
Numerous triggering factors that had an impact on child psychology during the pandemic [Triggering factors included compromised online pedagogy, financial issues in family, compromised life and wellbeing, home abuse that led to inclination towards committing crime, loneliness (being home‐confined), insomnia (sleeping disorder) and violence (physical abuse).].

About 57% participants in a study on 430 primarily non‐severe adolescent cases exhibited restricted daily activities at 176 ± 35.1 day mean follow‐up [96]. Age‐dependent immunological responses, expression of angiotensin‐converting enzyme 2, compromised blood‐brain barrier and social concerns influencing child behaviour (like school closures and social isolation) are some pathophysiological aspects linked to post‐COVID paediatrics. Long COVID associated with the biological and psychological being and possible risk factors and functionality of immune system in children were drawn [92]. More longitudinal research to clear it was necessary. Psychological impacts of the pandemic on children that had immediate and sustained implications are depicted in Figure 2.

Although the pandemic had numerous detrimental effects on child's life, it enhanced hygienic habits. A Nepali study evaluated altered water treatment, sanitation and hygiene (WASH) and impact on child health [97]. It found that the use of ceramic water filters rose from 12.2% to 34.8%, and there were indications of increasing chlorination in piped water‐supply systems. Significant improvements in hand‐washing (washing hands often, soap use and frequently doing it), and better kitchen and toilet hygiene were observed. Adopting these improved child health (like lower parasitic and respiratory infection rates, and diarrhea) greatly.

60 public primary schools in Addis Ababa, Ethiopia participated in a randomised control study to evaluate WASH‐related illness, absence and other educational outcomes during pandemic [98]. The study investigated the effects of urban school‐based WASH programme on child health and attendance through a random Project WISE. It was found, the probability of students reporting a respiratory ailment within the previous 7 days during the intervention follow‐up were 16% lower than those in control (*p* = 0.046). Also, menstrual care self‐efficacy increased slightly in the intervention group. The WHO defines telehealth (eHealth) as interventions, like mobile devices and applications and communication, which leveraged technology and enhanced wellbeing. Telemedicine (doctor‐patient communication via phone and internet) was crucial in pandemic and similar public health emergencies in pediatrics. This was critical particularly in transplant patient care, pediatrics surgical care, pediatric cardiology practice, pediatric weight management programmes, among others [99, 100, 101].

A study assessed the effect of pandemic on pediatric otolaryngology outpatient services using virtual outpatient clinics (VOPC) to leverage public access to heath during the pandemic [102]. According to the findings, telephone (185; 92.5%) was the most popular consultation method. In addition, absenteeism rates during pandemic were lower than those in conventional clinic during a comparable time‐frame before the pandemic. COVID‐19 exacerbated the needs of parenting, like low childcare and absence of in‐person education that caused physical and emotional strain, particularly for mothers. Walsh's theory served as foundation for longitudinal, mixed, qualitative study that looked at the resilience and family stress of mothers of 0–5 age‐group children. The particulars of pandemic mothering showed that while family resilience helped to understand reactions to systemic shock, increased workloads and decreased women participation in other activities were primarily responsible for the lack of the resilience [103].

Protective factors (*e.g*., supportive parenting, community interventions) during the pandemic, that buffered adverse effects, were also reported. A study on WASH practices was undertaken involving 363 families in Bataan Shipyard and Engineering Corporation (BASECO) property in Manila, a poor urban community in the Philippines [104]. The majority of families (237; 65.3%) drank distilled and purified water from water refilling stations in the community. Water boiling before drinking was prevalent among individuals with access only to tap water (146; 56.60%). Also, soap (356; 98.10%) was available and hand‐washing station (307; 84.57%) was accessible to the majority of the households. The majority knew that consuming raw or undercooked meat (298; 82.1%), use of contaminated water (301; 82.9%), incorrect food washing (309; 85.1%), and drinking untreated water (318; 87.6%) could lead to parasitic aliments.

Over 3300 remote families of native Alaska with no tap‐water were provided hand‐wash stations as miniature portable alternative sanitation system (Mini‐PASS) by US CDC during COVID‐19. 70% of families stated that they primarily used Mini‐PASS for handwashing [105]. Mini‐PASS's usefulness for other domestic duties was 51.4% (19 of 37) 3 months after intervention, and it was 77.8% (21 of 27) after twelve months. Awareness and practices at household in the Semen Bench district, Bench Sheko zone, southwest Ethiopia to prevent COVID‐19 were evaluated in a study [106]. Although 55% of participants understood COVID‐19 prevention well, only 48.5% practised these COVID‐19 prevention measures effectively. Prevalence of diarrhoea in children was 19.3%, and it was higher in households with inadequate COVID‐19 preventative measures. Further, it was seen that diarrhoea risk in children was 75.1% higher in households with poor COVID‐19 preventative practices than that with good practices (*p* = 0.004).

The impact of economic stress on maternal mental health on the newborn was assessed in a UK ‘COVID‐19 New Mum’ study [107]. It comprised 2031 moms whose children were born prior to the onset of COVID‐19. The odds of infants being fussy increased by 52% (OR = 1.52; 95% CI) and crying more increased by 64% (OR = 1.64; 95% CI) after controlling for covariates where the mother had inferior mental health.

### 3.10 Socioeconomic Factors

Socioeconomy was a critical determinant especially in sustained pandemic effects. Especially the girl child from financially‐poor background with unhygienic settings and the elderly had mental health risk, while children from higher socioeconomic strata were less vulnerable [108]. Data suggested that hard times during the pandemic increased the suicidal tendencies among children, adolescents and youth [109]. The emotional, social and psychological health among the severely affected seemed unending after pandemic, and distress among children and their parents continued. Such scenario among children of the healthcare workers specially was seemingly more severe, with significant mental health implications [110].

**Figure 3:**
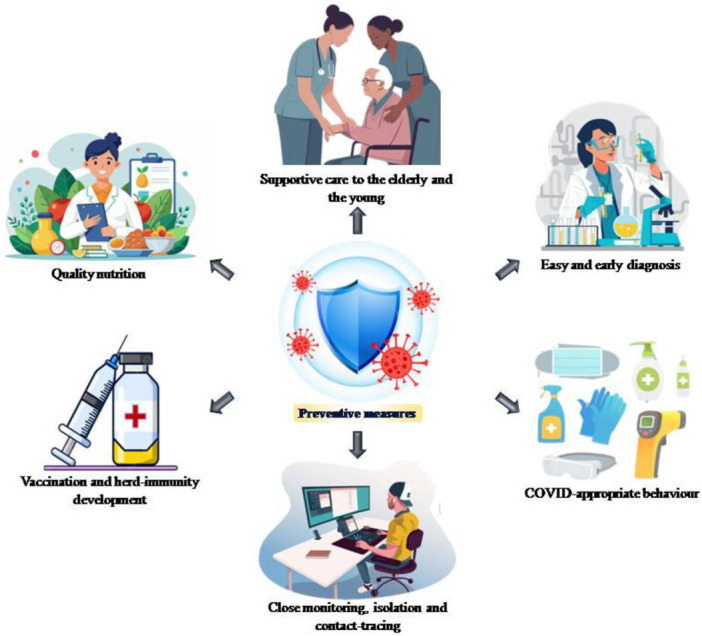
An illustration of preventive measures in the young to address COVID‐19 impacts.

Severity and long COVID in children were unclear unlike among adults as COVID‐19 infection reports were sparse [111]. Assessing and formulating a mechanism to decipher and address the impact in child that might affect individuals, society and the country was essential. Child mental health on anxiety, depression, attention deficit hyperactive disorder and irritability needed to be assessed [21]. Risk factors for mental health might be identified and correlated with emergency measures and psychological health. Effect of online pedagogy, long screen hours, academic outcome, and a link between school‐attendance and mental health also needed to be meticulously evaluated [112]. Figure 3 presents the measures to prevent or reduce the effect of pandemic on the young, including but not limited to quality nutrition, early diagnosis, vaccination, COVID‐appropriate behaviour, supportive care, rigorous monitoring, contact tracing and isolation. These interventions may vary across regions and locations based on the geographical, socioeconomic and available healthcare infrastructure considerations.

**Figure 4:**
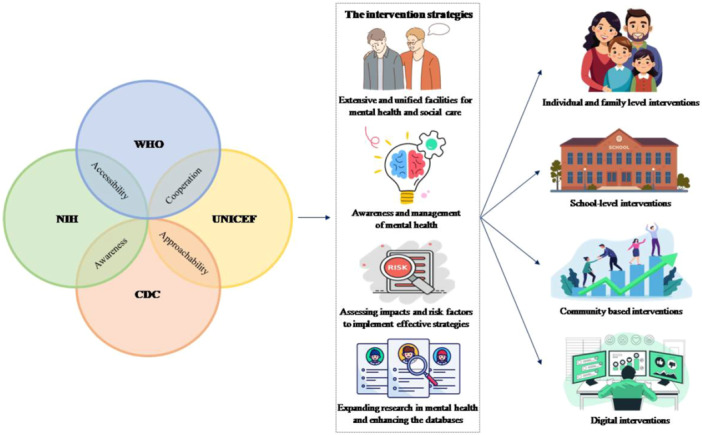
A schema illustrating the coordinated strategy by global health agencies for mental health management in children and adolescents.

Considering the difficulties faced by the economically deprived communities, an integrated strategy to address child mental health particularly among them was suggested (Figure 4). Agencies like UNICEF, WHO, CDC and the NIH could collaborate to expand and enhance databases on interdisciplinary research on mental health, optimal approachability and accessibility. This was likely to enhance awareness, build extensive and unified facilities for mental health and social care, management of mental health, assessing the risk factors and the probable impacts in implementing easily replicable and scalable effective strategies.

These envisaged interventions could be tried at individual, family, school and community levels, and also at the digital level (cyberspace). The challenges and opportunities in child mental health in the ‘new normal’ (post‐pandemic era) are new. Although international organisations extend support in numerous manners making great progress, targeted global initiatives particularly for vulnerable population in targeted pockets are awaited. The **supplementary file** details the global initiatives by agencies to tackle child mental health issues and recommendations for the economically‐deprived populations and yoga. By utilising cooperative, culturally aware and easily adoptable techniques, long‐term effects of pandemic on mental health of children and adolescents could potentially be reduced.

## 4 Discussion and Recommendations

Nutritional support is a crucial assurance to protect against viruses, enhance prognosis, treat and facilitate convalescence. Formulated whole nutritional food, in view of distinct clinical COVID‐19 symptoms, offers complete nutritional support with right nutrient combination. With essential macronutrients, micronutrients and vitamins, they effectively improve nutritional status and offer strong disciplined backing to improve survival and wellbeing. To increase body's defense against viruses and avoid infection, dietary supplementation in all age‐groups is suggested, including children, the elderly, and persons with co‐morbidities [113]. A study investigated the economic, lifestyle and nutritional impact of the pandemic on 9203 children aged 0.5–12.9 years and the parent/guardian in Malaysia, Indonesia, Thailand and Vietnam [114], and found that there was a major impact on children and the families. The impact of lockdown on children food intake had a relatively low effect across nations, but had a detrimental effect on food security particularly in Indonesia. Notably, lockdown led to healthier eating habits in Malaysia with fewer optional foods and among the vital food types. On the contrary, dairy consumption declined. As per self‐reporting, household intake of the majority of food types did not significantly change during lockdown elsewhere. Slightly reduced food intake during lockdown continued beyond lockdown in rural Thailand. Pandemic detrimentally impacted child's physical activity, employment security of families, and monthly household income across the globe.

A study evaluated and examined the magnitude of relationships between childhood malnutrition and morbidity in LMICs. According to findings, children with double‐burden malnutrition had a 5% higher morbidity risk although the difference was statistically insignificant [115]. On the other hand, malnourished children had 28% higher morbidity risk. Overweight children had a 29% lower morbidity incidence. Wasting was linked to a 1.1‐fold [RR: 1.094 (95% CI, 1.05–1.14)] higher morbidity risk, whereas children with double‐burden malnutrition and overweight children had lower morbidity risks [1.7%, RR: 0.983 (95% CI, 0.95–1.02) and 20%, RR: 0.80 (95% CI, 0.76–0.85)]. Severe acute malnutrition (SAM) among below 5‐year children in LMICs was caused by numerous factors including nutritional intake, health status, food, mother and child care, hygiene and sanitation, and healthcare access [116]. Prevalence of variables impacting stunting, underweight and concomitant wasting was 26.42% in LMICs. Underweight (27.6%), stunting (4.1%) and wasting (14.2%) were common in male. Also, stunting [adjusted odds ratio (aOR) = 1.52; 95% CI: (0.09, 0.89)] and underweight [aOR = 1.97; 95% CI: (0.01, 0.73)] in children were found to be associated to maternal education.

The association of the pandemic with anthropometric outcomes in 232,920 children under five in India was analysed using data from national family health survey (2019–2021) [117]. Compared to the pre‐COVID children, the age‐matched post‐COVID children (during 2020 and 2021) showed 1.2% higher underweight rates, 1.2% lower wasting rates, 0.1 lower height‐for‐age z‐scores (HAZ) and 0.04 lower weight‐for‐height z‐scores. Compared to age‐matched pre‐COVID children, post‐COVID children (during 2020) had 0.07 lower HAZ and 1.6%, 4.6% and 2.4% higher stunting, underweight and wasting rates, respectively. After early post‐pandemic decline, results showed rebound tendency in child anthropometric outcomes in 2021. While funds were immediately needed to reduce child hunger and enhance child health outcomes, health and food systems need to be more resilient to COVID‐19 like emergencies. Child and teenager mental health was most impacted by the pandemic. In a study involving 293 parents of children in 4–6 age‐groups, the aggressive, anxious and resilient traits of children and families were evaluated [118]. The findings indicated that the aggressive tendencies in preschoolers during the pandemic were influenced by parental resilience and worries. The findings also indicated that supporting the development with more precise techniques and interventions in early life was critical for the mental health of children, particularly during pandemic‐like crisis situations.

Some were found to develop Long COVID syndrome, the post‐acute sequelae of COVID‐19 (PASC) or the post‐COVID‐19 condition (PCC), a chronic sickness lasting 3 months or long after SARS‐CoV‐2 infection [119]. Symptom or sign that might improve, worsen or remain unaltered indicate Long COVID. Some underlying long‐COVID symptoms are mitochondrial dysfunction (reduced cellular energy synthesis), endocrine disruptions (altered hormonal balance and metabolism), pulmonary effects (persistent lung inflammation and mood), immune dysregulation (autoimmunity and hyperinflammation), and vascular dysfunction (reduced blood flow and possible micro‐clots). Also, there could be psychological stress (anxiety and stress from infection and isolation) facilitated through autonomic nervous system dysfunction (altered heart rate, blood pressure and digestive responses), and neurological (direct brain and nerve) issues. Children and teenagers experienced chronicity related to respiratory, otorhinolaryngological, cardiovascular, neurological, gastrointestinal and metabolic conditions. The impacted children were more likely to experience behavioral, psychological and mental health issues. Further, variables like age, severity of first infection, and viral variant impacted long COVID prevalence in children. 1%–45% of kids with a COVID‐19 history may have symptoms that lasted for months [120]. Also, those with severe initial infections or pre‐existing medical conditions (comorbidity) were more likely to acquire long COVID, and older children and adolescents were more vulnerable than the younger ones.

A study examined the evidence of long‐COVID in children and teenagers. The findings showed a comparatively steady long COVID trend whose prevalence declined significantly over time [121]. Teenagers were more frequently afflicted than the younger ones, and prevalence of long COVID seemed to vary among various pediatric age‐groups. Data indicated that children and adolescents were less frequently impacted by long COVID as compared to adults. Long COVID was linked to an array of symptoms and indicators that impacted nearly every organ in children and adolescents, with the respiratory, cardiovascular and neuropsychiatric systems being most frequently impacted. SARS‐CoV‐2 infected children in 6–17 age‐groups were included in a multi‐location longitudinal observational cohort study recruiting participants from above 60 US community healthcare centres [122]. It compared the differences in long COVID symptoms between children aged 6–11 and adolescents aged 12–17. The results showed that the median time between infection and development of long COVID symptoms was 506 days for 6‐11 age‐group children and 556 days for adolescents of 12–17 years. It was seen that school‐age children more likely experienced neurological and cognitive dysfunction, pain and gastrointestinal issues than teenagers who were more likely to experience altered smell or taste, pain and symptoms associated to weariness or malaise.

1129 children with SARS‐CoV‐2 infection were evaluated for long COVID 1–3 months, 3–6 months and 6–12 months after infection in an Italian study [123]. It was found that 16.2% children had long COVID and 68.6% had at least one post‐COVID symptom. Respiratory issues (43.4%), neurological and cognitive dysfunction (27.7%), gastrointestinal symptoms (22.1%), exhaustion (21.6%) and sleep disruptions (18.8%) were most commonly encountered. Prevalence of cardiovascular, neurological and cognitive impairment was significantly high in older children and females. 141 children and adolescents hospitalised with COVID‐19 and 141 controls who were unaffected participated in an Iranian cohort study to compare children and adolescents with the control group to assess long COVID [124]. The average age of hospitalised children with COVID‐19 was 79 ± 5.24 months, 57.4% being male. 46 children (32.6%) in the COVID‐19 group had prolonged COVID symptoms. Fatigue was most commonly seen (54.3%), followed by symptoms of depression or anxiety (34.7%) and focus or concentration issues (41.3%). 65.2% of the hospitalised kids with chronic COVID‐19 symptoms had moderate illness level. The severity significantly correlated with joint and muscle discomfort (*p* = 0.025). Weight loss (*p* = 0.047), feelings of anxiety or despair (*p* = 0.008), and hospitalisation duration and coughing (*p* = 0.022) were observed. A history of smoking or being around smoke (OR = 12.45, CI = 3.14–49.36) and the elderly were found to be long COVID risk factors.

Thoughtful non‐pharmacological therapeutic interventions included trauma‐focused cognitive behavioral (TF‐CBT), cognitive processing (CPT), and prolonged exposure (PE) therapies. Including parent/caregiver, TF‐CBT and evidence‐based therapies helped a child or adolescent overcome trauma [125]. It is a phasic therapy that established novel safety skills, processing the traumatic memory, and controlling the symptoms. PRACTICE (psychoeducation, parenting, relaxation, affective modulation, cognitive coping, trauma narration and cognitive processing), mastering trauma reminders *in vivo*, conjoint child‐parent sessions and enhanced safety were frequently used to summarise component‐based therapy model. Post‐trauma stress disorder (PTSD) could be treated with CPT, a cognitive‐behavioral therapy. By engaging to recognise, examine and reframe ‘trapped/jammed points’ in thoughts, individuals are assisted to challenge and alter negative thoughts around a traumatic incident. Self‐worth, trust and a feeling of security and control could enhance. CPT is an evidence‐based twelve sessions per week treatment.

There are two different approaches especially for mental health disorders like PTSD, prolonged exposure (PE) and mindfulness‐based therapies to improve results. While mindfulness promotes nonjudgmental awareness of the present, PE entails facing trauma memories and triggers. They could assist with PE to control the anxiety during exposure and mindfulness to avoid being overwhelmed by the past and stay in the present [126]. PTSD to assess and treat is recommended by the National Institute for Health and Care Excellence (NICE) guidelines in the UK for children, adolescents and adults [127]. Individuals with PTSD or at high risk of acquiring PTSD (like post‐COVID‐19 hospitalisation) are thoroughly evaluated on physical, psychological and social needs following the NICE guidelines. NICE suggests eight to twelve 90‐min sessions of individual TF‐CBT that included PE therapy, CPT, cognitive therapy for PTSD, and narrative exposure therapy (NET). NICE also supports eye movement desensitisation and reprocessing (EMDR) therapy.

Digital‐based interventions to treat mental illness are being more frequently used, especially to treat trauma‐related disorders due to the pandemic [128]. The terms ‘e‐Health’, ‘e‐Mental Health’, and ‘e‐Therapy’, delivered through various techniques, is used interchangeably for digital‐based interventions. These include the internet, telephone, computers, mobile and other web‐based and online deliverables. Additionally, digital‐based interventions give an option to receive supported or self‐guided therapies. While supported interventions vary in terms of who offered the support, how much support was needed, and the modality used (*e.g*., video‐conferencing, phone call, text/chat functions and email), self‐guided interventions may include the assistance of automated systems (*e.g*., notifications and prompts). An at‐a‐glance summary of the concern‐wise evidence strength is provided in Table 2 to highlight key findings for the benefit of the readers. However, an extended summary can be found in Table S3 (**Supplementary File**).

**Table 2:** A summary of the concern‐wise strength of evidence.

Concern	Key supporting factors	Limitations	Strength of evidence
Lockdowns effect	Multiple cross‐sectional studies; consistent findings across geographic regions; documented increases in anxiety, depression, and behavioural changes	Reliance on parent‐reported outcomes; limited longitudinal data; variability in lockdown duration and intensity across studies	Robust evidence supports the role of lockdowns (**moderate to strong**)
Food habits and nutrition	Well‐documented weight gain patterns; consistent findings across multiple nations (Poland, Saudi Arabia, Britain, Turkey, Italy); clear associations with rise in high‐calorie food consumption	Limited data on long‐term nutritional outcomes; self‐reported dietary data subject to bias	Robust evidence supports the effect of food intake patterns (**strong**)
Impact on children in LMICs	Multiple studies from diverse LMICs; documented disruptions in healthcare access and education	Heterogeneous study designs; limited controlled studies; varying pandemic response measures across countries	Emerging evidence suggests the effect of living conditions (**moderate**)
COVID‐19 vaccination in children	Large‐scale clinical trials; regulatory approval data; extensive safety monitoring	Limited long‐term safety data; evolving evidence on variant‐specific efficacy; ongoing debate about necessity in healthy children	Robust evidence supports the vaccination concern (**strong**)
Long COVID in children	Multiple studies documenting persistent symptoms; some controlled trials comparing infected versus non‐infected children	Highly variable prevalence estimates (0%–67% at 8–12 weeks; 8%–51% at 6–12 months); inconsistent case definitions; lack of standardised diagnostic criteria	Emerging evidence suggests sustained health effect of COVID (**weak to moderate**)
Socioeconomic disparity	Consistent findings across studies showing greater impact on low‐income families; documented variation in access to healthcare, education and nutrition	Difficulty separating pre‐existing disparities from pandemic‐specific effects; limited intervention studies	Robust evidence supports the impact of socioeconomic status (**moderate to strong**)
Telehealth effectiveness	Multiple studies show feasibility and acceptance; reduced absenteeism in pediatric services	Limited data on clinical outcome compared to in‐person care; selection bias in participants	Robust evidence supports the digital health interventions (**moderate**)

### 4.1 Limitations of the Study

Literature on experimental studies or research articles on the impact of pandemic on children are limited, which potentially limits the depth of human understanding. Further, as studies from varying geography and demography reported conflicting findings, there is a potential study bias which could affect the generalisability of the findings. Inclusion of the articles published only in English could lead to analyses bias, as the literature in other languages might provide additional insights or different perspectives, so also the preprints or unpublished data. Most included studies are observational and survey‐based, limiting causal inference. This narrative review is broad, less structured and summarises an array of findings. The conclusions are hypothesis‐generating, not definitive. Although this review may have bias, it thoroughly and thoughtfully synthesises the literature to identify prevalent knowledge and the gaps for reliable and validated findings. However, focused systematic review is suggested that could minimise bias. Thus, the need for longitudinal studies focused on each specific aspect is recommended. This narrative review would help evaluate the strength of the presented evidences as it carried out a quality check of the literature to ascertain the methodological rigor and potential biases.

## 5 Conclusion

The available data on prolonged effect of pandemic in children was limited, inconsistent and was found to be unorganised or of low calibre. As the precise prevalence of disorders is unknown yet, discerning between the actual post‐acute COVID‐19 scenario and the effects of social restrictions was challenging. Excellently organised multidisciplinary research designs with spread and depth to determine best possible interventions for acute illnesses was necessary. It may help mobilise resources to combat long‐COVID, and help nullify the overall detrimental impacts on children and adolescents. Studies adopting extended defined COVID research as offered by the WHO could be encouraged to ensure data harmonisation and homogeneity. Controlled clinical investigations as against the questionnaire‐based surveys may be preferred to provide unbiased results of the true prevalence and sustained ‘COVID‐19’ sequelae in children. Further, the effect of novel viral variants and the prevailing long COVID needs insights to help healthcare systems allocate the resources promptly and appropriately. Global community must concentrate on the areas of specific action and invest accordingly to successfully combat the devastating long‐term impact of pandemic, especially among the young. Governments could ensure feeding the children for their health and wellbeing, and easy access of the most‐critically affected communities and healthcare workers to the supplied protective gears. Also, preserving the access to life‐saving medication and vaccines, maternal and infant care, and child health services were crucial.

Rather than as definitive, the seminal evidences and findings collated in this study clearly position the article as hypothesis‐generating. Analysing the select literature and findings (strength of evidence) in those, certain credible conclusions were drawn and recommendations deduced based on the commonalities therein. The article has synthesised the main findings across nutrition, pedagogy and mental health in the younger age groups. There were stronger evidences related to factors like role of lockdowns, effect of food intake patterns, vaccination concern, impact of socioeconomy, and the digital health interventions (as robust evidences), and moderate evidence like effect of living conditions, and health effect of long‐COVID (as emerging evidences). The findings and the generated hypotheses emphasises on implications of the effect of COVID‐19 in particular and pandemics in general on the children and adolescents, and paves the way for a modified and more effective clinical practice and public policy.

An integrated strategy considering the difficulties faced by specific deprived communities is suggested to address child mental health especially within economically deprived communities. Targeted initiatives especially within the susceptible population were awaited, although constant support in innumerable ways was being extended by many across the globe in isolated pockets. Sustained impact of pandemic on the mental health of children and adolescents could be reduced potentially through cooperative, culturally sensitive and easily adoptable strategies. The findings of this narrative review could be suitably applied in similar lines. Lastly, joint efforts across the healthcare and wellbeing department and agencies at every stage across the globe with children and the adolescents at the core shall be beneficial and safeguard the future generation in the face of a pandemic. Further, necessary concerted and collaborative scientific investigations are recommended.

## Author Contributions

**Ranjan K. Mohapatra:** conceptualization, supervision, writing – review and editing, visualization. **Ashish K. Sarangi:** data curation, writing – original draft. **Venkataramana Kandi:** writing – original draft, validation. **Hayam A. Alrasheed:** writing – original draft, validation. **Ali A. Rabaan:** writing – original draft, validation. **Md. Kudrat‐E‐Zahan:** writing – original draft, data curation.

## Funding

The authors received no specific funding for this work.

## Ethics Statement

This article does not require any human/animal subjects to acquire such approval.

## Conflicts of Interest

The authors declare no conflicts of interest.

## Transparency Statement

The lead author Ranjan K. Mohapatra, Snehasish Mishra, Md. Kudrat‐E‐Zahan affirms that this manuscript is an honest, accurate, and transparent account of the study being reported; that no important aspects of the study have been omitted; and that any discrepancies from the study as planned (and, if relevant, registered) have been explained.

## Supporting information

R4 SI file Children 12 2 26.

## Data Availability

All the data in this review are included in the manuscript. No new data is generated.
