# Supplementary material for: Critical Evaluation of the Impact of COVID‐19 Pandemic on Child Health and Wellbeing and Suggested Preparedness for Future Pandemics–A Narrative Review
Source: Health Sci Rep. 2026 Mar 17;9(3):e72073. doi: 10.1002/hsr2.72073 (PMC13098172; doi:10.1002/hsr2.72073)
Supplement: Supplementary file 1 — R4 SI file Children 12 2 26. [file HSR2-9-e72073-s001.docx]

**Supplementary file**

**Critical evaluation of the impact of COVID-19 pandemic on child health and wellbeing and suggested preparedness for future pandemics – A narrative review**

**Table S1.** Search strategies used in Scopus database

| No. | Keywords | Synonyms | Total |
| --- | --- | --- | --- |
| 1 | COVID-19 | TITLE-ABS-KEY (“COVID-19” OR “SARS-CoV-2”) | 290,759 |
| 2 | Child | TITLE-ABS-KEY (“Child*” OR “Infant*” OR “Adolescent*” OR “Pediatrics”) | 3,255,163 |
| 3 | Child health | TITLE-ABS-KEY ("Child health" OR "Mental health" OR "Wellbeing" OR "Quality of life") | 1,079,182 |
| 4 | Pandemic preparedness | TITLE-ABS-KEY ("Pandemic prepared*" OR "Emergency prepared*" OR "Health system resilience") | 13,281 |
| 5 | #1 AND #2 AND 3# AND #4 | TITLE-ABS-KEY (“COVID-19” OR “SARS-CoV-2”) AND (“Child*” OR “Infant*” OR “Adolescent*” OR “Pediatrics”) AND ("Child health" OR "Mental health" OR "Wellbeing" OR "Quality of life") AND ("Pandemic prepared*" OR "Emergency prepared*" OR "Health system resilience") | 84 |

**Table S2.** Search strategies used in Web of Science database

| No. | Keywords | Synonyms | Total |
| --- | --- | --- | --- |
| 1 | COVID-19 | TS = (“COVID-19” OR “SARS-CoV-2”) | 310,75 |
| 2 | Child | TS = (“Child*” OR “Infant*” OR “Adolescent*” OR “Pediatrics”) | 2,975,163 |
| 3 | Child health | TS = ("Child health" OR "Mental health" OR "Wellbeing" OR "Quality of life") | 1,279,141 |
| 4 | Pandemic preparedness | TS = ("Pandemic prepared*" OR "Emergency prepared*" OR "Health system resilience") | 13,281 |
| 5 | #1 AND #2 AND 3# AND #4 | TS = (“COVID-19” OR “SARS-CoV-2”) AND (“Child*” OR “Infant*” OR “Adolescent*” OR “Pediatrics”) AND ("Child health" OR "Mental health" OR "Wellbeing" OR "Quality of life") AND ("Pandemic prepared*" OR "Emergency prepared*" OR "Health system resilience") | 71 |

**The benefits of yoga (bhramari pranayama)**

In this technologically advanced society, the human world is moving crazily fast. It is imperative to maintain focus and attention while adjusting oneself, which could be attained through Yoga. Frequently misunderstood as a religious form, Yoga is actually a scientifically-sound discipline. The Sanskrit meaning of yoga is ‘to unite’ which means to link or balance the mind and the body. It believes that a happy and contented life could be attained if correct breathing, appropriate exercise and right thoughts are practised. This became even more essential during the pandemic [1]. So what is the appropriate age to practice Yoga? The magical age group is 6-8 years [2]. Yoga is now an essentiality in Indian schools, many grouping it with physical education classes as ‘games period’. [3] Taught in its pure context, Yoga could ensure raising a crop of peaceful, cheerful and very adaptable kids. Pandemic-related anxiety and sadness in the young could lead to stress that could disrupt physiological balance and alter homoeostasis in the body ultimately leading to mental sickness. Cortisol is produced in excess on the HPA axis with an increase in stress. Yoga manages well the cortisol levels and depression both. Stress could trigger both inflammatory cytokines production and inflammatory response [2]. IL-10 down-regulates or inhibits the expression of numerous pro-inflammatory cytokines, including IL-1*β*, making it a potent anti-inflammation promoter [4]. Yoga reduced proinflammatory cytokines and elevated anti-inflammatory cytokines. It also reduced oxidative stress [5]. Altered inflammatory mediators through yoga practice is known. Thus, Yoga could address stress pathophysiology and promote mental health. Yoga additionally increases circulation of body fluids, stabilises autonomic nervous system, strengthens the muscles, enhances endurance and increases resilience in children.

Practisingbhramari pranayama among teenagers is one such case instance [5]. Schoolchildren were trained bhramari pranayama for six months, and were frequently monitored. Scientifically designed, numerous health indicators were analysed. It was found that cardiovascular parameters (heart rate and variability), blood pressure, pulmonary function and cognitive functions improved. The benefits as revealed by research on cardiovascular and cognitive functioning was attributed to the dominant parasympathetic nerves and increased cerebral blood flow [6,7]. To reach to the enormously large susceptible young population, it is strongly advocated to consider Yoga as a curriculum. For extended coverage and quick access especially in this ‘new normal’ era where social distancing is paramount, existing technological platforms could be used to full potential for Yoga instructions.

**The initiatives by the global agencies to address child mental health issues**

COVID-19 is considered an abnormally critical health issue as a dangerously powerful health and economy destroyer of this century worldwide. Although it had a clinically low effect on the young than the adults, the children were incidentally influenced in numerous ways both physically and mentally. There were increasing reports of less physical activities, obesity (weight gain), increased screen-time on the mobile, computer and television, and sleep disorders during the lockdown which are bound to have short- and long-term implications on the physical and mental as well as psychological health. In order to prevent the short- and long-term sequel, such adverse situation that has built-up needs highlighting and timely interventions. Adolescence phase of one’s life is critical where appearance and peer approval are key values during this developmental period, and studies have linked obesity and mental health in adolescents. An increasing risk of psychiatric disorder leading to depression is perceived due to the obesity-related high level of dissatisfied appearance, inferior self-esteem and the perception of being stigmatised. In this light, there have been a few recent initiatives by global agencies to counter such a crisis that is building up.

Experiencing adversity during the early stage of one’s life is a well-recognised risk factor in mental disorder development. Mental health is an essential component of health and wellbeing as stated in the constitution of the WHO while defining health, as ‘*health is a state of complete physical, mental and social wellbeing and not merely the absence of disease or infirmity*’[8]. The factors influencing mental health and its disorders extend beyond traits like managing one’s thoughts, emotions, behaviour and social interactions (an individual’s internal factors), to include social, cultural, economic and environmental aspects (the external factors). These encompass national policies, social protection, standard of living, work environments and the community support networks [9].

Agencies like WHO are at the forefront, especially in the wake of the COVID-19 pandemic, to address the global child mental health issues. The WHO and the OHCHR have promoted a human rights approach to mental health. Recognising the profound impact of the pandemic on the mental wellbeing of children, WHO has launched a comprehensive mental health action plan[10]. Updated and revamped in 2021 at the 74^th^ World Health Assembly, the Comprehensive Mental Health Action Plan 2013–2030 of the WHO calls on nations to align their mental health-related legislation with the 2006 CRPD and other international and regional human rights treaties, It urges nations to alter and/or revoke stigma, discrimination and human rights violation supporting laws. The action plan includes practical goals such as, 1) stepping-up effective leadership and governance for mental health, 2) providing comprehensive, integrated and responsive community-level mental health and social care services, 3) implementing strategies to promote mental health, and 4) revamping the information systems and evidence-based mental health research [10]. The plan further extends to the integration of mental health support in primary healthcare thereby making it easily accessible to children across communities. WHO has developed guidelines for parents and caregivers to maintain child mental health specifically during and after the pandemic. While acknowledging the challenges posed by isolation and disrupted routines, the guidelines emphasise on the significant role of a nurturing and supportive environment for children [11]. WHO also collaborates with educational institutions for the promotion of mental health literacy among children and adolescents. The approach also includes public awareness campaigns to destigmatise mental health issues among children and adolescents to encourage open conversations and early interventions.

As per data of the UNICEF, one in every seven children and adolescents is affected by mental disorders [12]. Most mental health disorders originate early in life, with 50% arising before the age of 14 and 75% by the mid-20s. Regardless of the high burden and early onset, most mental health conditions often remain untreated and unrecognised. Kohn *et al.* (2004) estimated that 70% of the population in the age group of 15 and above living with mental health conditions lack access to adequate care [13] and that this gap is even higher in the LMICs, which has high children and young people residing[14]. Owing to the mental health issues being largely critical in children, UNICEF took up a holistic approach to address it [15]. The strategy involves a synergy between direct intervention and systemic changes. UNICEF funds and supports parents and family-based, school-based and community-based as well as digital programmes based intervention strategies in numerous countries to provide psychological counselling and support to the pandemic affected children. Out of these, the school-based initiatives seemed to have significantly improved the wellbeing of kids and teenagers by reducing anxiety and depression in them and enhancing their coping mechanisms [16,17]. The programmes are designed to be more accessible to children from diverse backgrounds while promoting especially in a community settings by making them culturally sensitive [18-20]. Additionally, psychoeducation, parent and family skills trainings, and behavioural, psychosocial and trauma-focused cognitive behavioural therapy are examples of parent and family-focused interventions with better parenting and family dynamics which can be beneficial for the mental health of a child [21]. The data obtained during the early COVID-19 crisis suggests that mental health issues were worse during the pandemic as a result of lockdown, school-closure and the global economic downturn, putting a child at psychosocial distress like anxiety, depression and externalizing behaviour [22,23].

The UNICEF systematically advocates for policies to create child-friendly environment. The urgency to increase research funding for mental health intervention and significance of targeting specific risk factors across age groups in LMICs is felt. The UNICEF has reinstated its commitment towards funding and safeguarding the mental health of children and adolescents. Study estimates that 90% of the research on child and adolescent mental health has been done in high-income nations, and the data from low-resource settings is low. The UNICEF and WHO have joined hands to support at global scale with a collective vision of ‘*By 2030, children and adolescents living in countries targeted under the Joint Programme will experience reduced suffering and improved mental health and psychosocial wellbeing and development*’[24]. The strategy is to collectively address challenges that include the leadership crisis, investment and coordination across sectors and line departments, poor frontline capacity, insufficient focus on promoting mental wellbeing and addressing the associated stigma, and the lack of data and evidence on what works [24].

The NIH and the CDC in the US have undertaken significant initiatives to tackle child mental health issues. The NIH has funded extensive research to understand COVID-19 as it impacted child mental health, with a focus on developing evidence-based interventions [25]. A comprehensive survey-based study by Novins*et al*. (2021) identified the key research priorities in adolescent mental health during the pandemic. It gathered opinions from child development professionals and paediatric mental health experts, highlighting concerns about increased stress in families, the the pandemic as it impacted the child and adolescent mental health. Key research priorities as identified by the survey participants included a) documenting impacts, risk and protective factors like mental health, cognitive development, susceptible children and an access to medical service, and b) developing and testing community, hospital and school-level interventions [25]. A recent article discussed several NIH programmes focused on child mental health research those include ECHO, ABCD, HBCD and MIRA [26]. The nationwide ECHO programme aimed to study early environmental impacts on child health and development, whereas ABCD focused on understanding how the childhood experiences shaped the brain and emotional development in the 9-10 age-group into early adulthood. HBCD aimed at examining similar aspects in a younger cohorts starting from 2^nd^ trimester of pregnancy to the age of 10 years. Although NIH supports investigator-initiated projects through R01 grants, it encourages researchers to consider MIRA from the NIGMS. These initiatives collectively highlight the importance of understanding various factors that impact child neurodevelopment that play a direct role in child mental health interventions.

The CDC developed comprehensive resources for parents, educators and healthcare providers for social, emotional and mental wellbeing of young COVID-19-affected children. These include guidelines to recognise mental health issues in child, tips to support children during the pandemic and information on the modalities to access mental health services[27-29]. The CDC researchers discovered that teenagers had mental health issues earlier than the pandemic outbreak, and the cases worsened by the pandemic that disrupted their schools, kept them away from their classmates, raised their stress levels, and restricted their access to care. The results of a survey on the experiences of high-school students during the pandemic revealed that 44% of respondents felt depressed or hopeless often, 37% most of the time or always experienced poor mental condition, 20% contemplated committing suicide, and 9% had in fact tried it [30]. This resulted in the CDC urging schools to support students' access to resources and to build a sense of community. Schools were suggested to enhance staff training, foster better family ties, encourage inclusion, and incorporate social and emotional learning mechanisms.

**Suggestions and recommendations for the economically-deprived populations**

Improved accessibility to mental health services is very crucial. It can be incorporated into the existing community health centres and schools as these are frequently visited by families with lower income. Remote communities can also be greatly aided through the far-reaching telehealth services and mobile health units. Training in psychological first aid and basic mental health care is another important component that could be imparted to the community health workers and educators. As dependable community members, these people could act as the primary point of contact for kids and families in need of mental health assistance. In addition, it is critical to address the more general social mental health determining factors like housing, education and poverty.

Mental health interventions should be developed in consultation with community members to ensure they are culturally appropriate and resonate with the community's values and beliefs. Governments, non-governmental organisations and local communities working hand-in-hand actively might reduce the increased stress due to poverty and foster a positive atmosphere. Community engagement and culturally sensitive approaches are important additional determinants. Peer support programmes involving adolescents and young adults from the community can also be effective to provide relatable support to children and encourage them to seek help.

**Table S3.** Summary of Evidence Strength by Topic Area

| **Topic Area** | **Strength of Evidence** | **Key Supporting Factors** | **Limitations/Uncertainties** |
| --- | --- | --- | --- |
| Lockdown Effects on Mental Health | Moderate to Strong | Multiple cross-sectional studies; consistent findings across geographic regions; documented increases in anxiety, depression, and behavioral changes | Reliance on parent-reported outcomes; limited longitudinal data; variability in lockdown duration and intensity across studies |
| Food Habits and Nutrition | Strong | Well-documented weight gain patterns; consistent findings across multiple countries (Poland, Saudi Arabia, Britain, Turkey, Italy); clear associations with increased consumption of high-calorie foods | Limited data on long-term nutritional outcomes; self-reported dietary data subject to bias |
| Impact on Children in LMICs | Moderate | Multiple studies from diverse LMICs; documented disruptions in healthcare access and education | Heterogeneous study designs; limited controlled studies; varying pandemic response measures across countries |
| Online Education Impact | Moderate | Consistent reports of increased screen time; documented educational disruptions affecting 1.6 billion children globally | Limited data on actual learning outcomes; lack of standardized assessment tools; confounding by socioeconomic factors |
| Child Maltreatment | Moderate | Some longitudinal data (e.g., China study with 8-month follow-up); documented increases in sexual abuse post-lockdown | Conflicting findings across studies; underreporting bias; difficulty separating lockdown effects from other pandemic factors |
| Sleep Disturbances | Moderate to Strong | Multiple studies with consistent findings; age-specific patterns documented (34% in 2020, 27% in 2021 for older children) | Primarily survey-based; limited objective sleep measurements |
| COVID-19 Vaccination in Children | Strong | Large-scale clinical trials; regulatory approval data; extensive safety monitoring (e.g., Canadian cohort study with 131,032 two-dose recipients) | Limited long-term safety data; evolving evidence on variant-specific efficacy; ongoing debate about necessity in healthy children |
| Vaccine Side Effects | Strong | Well-documented through systematic reviews; clear patterns (myocarditis more common in males, primarily after second dose); specific incidence rates available | Relatively short follow-up periods; may underestimate rare adverse events |
| Long COVID in Children | Weak to Moderate | Multiple studies documenting persistent symptoms; some controlled trials comparing infected vs. non-infected children | Highly variable prevalence estimates (0-67% at 8-12 weeks; 8-51% at 6-12 months); inconsistent case definitions; lack of standardized diagnostic criteria |
| Multisystem Inflammatory Syndrome (MIS-C) | Strong | Well-characterized clinical syndrome; multiple case series; clear temporal relationship with COVID-19; documented cardiovascular involvement | Relatively rare condition; limited understanding of long-term outcomes |
| Socioeconomic Disparities | Moderate to Strong | Consistent findings across studies showing greater impact on low-income families; documented differences in access to healthcare, education, and nutrition | Difficulty separating pre-existing disparities from pandemic-specific effects; limited intervention studies |
| Screen Time Increase | Strong | Multiple studies with consistent findings; documented association with behavioral and sleep problems; clear dose-response relationship | Primarily correlational data; confounded by lockdown restrictions |
| Impact on Children with Comorbidities | Moderate | Multiple case series and cohort studies; documented increased vulnerability | Heterogeneous patient populations; limited controlled studies; variable definitions of comorbidities |
| WASH (Water, Sanitation, Hygiene) Improvements | Moderate to Strong | Documented improvements in hand washing practices; measurable reductions in diarrheal diseases (Ghana study: p<0.001); improved health outcomes in Nepal study | Limited to specific geographic areas; sustainability of behavior changes uncertain |
| Telehealth Effectiveness | Moderate | Multiple studies showing feasibility and acceptance; reduced absenteeism rates in pediatric services | Limited data on clinical outcomes compared to in-person care; selection bias in participants |
| Maternal Mental Health Impact on Infants | Moderate | UK study (n=2031) showing 52% increased odds of infant fussiness and 64% increased crying with worse maternal mental health | Cross-sectional design; potential bidirectional effects |

**Interpretation and Implications**

**Strong Evidence Areas:** Topics with strong evidence (COVID-19 vaccination efficacy and safety, food habit changes, screen time increases, MIS-C characteristics) provide reliable foundations for policy recommendations and clinical practice guidelines. These areas benefit from large sample sizes, consistent findings across geographic regions, and robust study designs including controlled trials.

**Moderate Evidence Areas:** Topics with moderate evidence (lockdown mental health effects, educational impacts, socioeconomic disparities) show generally consistent trends but lack the methodological rigor or long-term follow-up needed for definitive conclusions. These areas warrant continued monitoring and research with improved study designs.

**Weak/Uncertain Evidence Areas:** Long COVID in children represents the primary area of uncertainty, with highly variable prevalence estimates and inconsistent findings. This reflects the challenges of defining, diagnosing, and measuring persistent symptoms in pediatric populations. Standardized diagnostic criteria and prospective longitudinal studies are urgently needed.

**Geographic Considerations:** Evidence strength varies by geographic region, with stronger evidence from high-income countries (United States, United Kingdom, Europe) compared to LMICs. This disparity highlights the need for expanded research in underrepresented populations.

**Methodological Considerations:** Most evidence comes from observational studies and surveys rather than randomized controlled trials, which limits causal inference. The rapid evolution of the pandemic, emergence of new variants, and changing public health measures created additional challenges for longitudinal research and comparison across time periods.

**References:**

1. Basu-Ray I, Metri K, Khanra D, et al. A narrative review on yoga: a potential intervention for augmenting immunomodulation and mental health in COVID-19. *BMC Complement Med Ther* 2022;22(1):191.
2. Marconcin P, Werneck AO, Peralta M, et al. The association between physical activity and mental health during the first year of the COVID-19 pandemic: a systematic review. *BMC Public Health* 2022;22(1):1-14.
3. Nagarathna R, Kumar S, Anand A, et al. Effectiveness of Yoga Lifestyle on Lipid Metabolism in a Vulnerable Population-A Community Based Multicenter Randomized Controlled Trial. *Medicines (Basel)* 2021;8(7):37.
4. Slavich GM, Irwin MR. From stress to inflammation and major depressive disorder: a social signal transduction theory of depression. *Psychol Bull* 2014;140(3):774-815.
5. Gupta S, Gautam S, Kumar U, et al. Potential Role of Yoga Intervention in the Management of Chronic Non-malignant Pain. *Evid Based Complement Alternat Med* 2022;2022:5448671.
6. UN Sustainable Development Group, <https://unsdg.un.org/sites/default/files/202004/160420_Covid_Children_Policy_Brief.pdf>Accessed January 11, 2024.
7. Kuppusamy M, Ramaswamy V, Shanmugam P, Ramaswamy P. Yoga for children in the new normal–experience sharing. *Journal of Complementary and Integrative Medicine* 2021;18(3):637-40.
8. Constitution of the World Health Organization. https://www.who.int/about/accountability/governance/constitution. Accessed January 11, 2024.
9. Mental health, human rights and legislation: guidance and practice. https://www.who.int/publications/i/item/9789240080737. Accessed January 11, 2024.
10. Comprehensive Mental Health Action Plan 2013-2030. https://www.who.int/publications/i/item/9789240031029. Accessed January 11, 2024.
11. The impact of COVID-19 on mental health cannot be made light of. https://www.who.int/news-room/feature-stories/detail/the-impact-of-covid-19-on-mental-health-cannot-be-made-light-of. Accessed January 11, 2024.
12. MIND THE GAP: Child and Adolescent Mental Health and Psychosocial Support Interventions – An evidence and gap map of low and middle-income countries. https://www.unicef-irc.org/publications/1589-mind-the-gap-child-and-adolescent-mental-health-and-psychosocial-support-interventions-an-evidence-and-gap-map.html. Accessed January 11, 2024.
13. Kohn R, Saxena S, Levav I, Saraceno B. The treatment gap in mental health care. *Bull World Health Organ* 2004;82(11):858. doi:/S0042-96862004001100011
14. Kieling C, Baker-Henningham H, Belfer M, et al. Child and adolescent mental health worldwide: evidence for action. *Lancet* 2011;378(9801):1515-1525.
15. Mental health and well-being | UNICEF Parenting. https://www.unicef.org/parenting/mental-health. Accessed January 11, 2024.
16. Barry MM, Clarke AM, Jenkins R, Patel V. A systematic review of the effectiveness of mental health promotion interventions for young people in low and middle income countries. *BMC Public Health* 2013;13(1):835.
17. Bradshaw M, Gericke H, Coetzee BJ, et al. Universal school-based mental health programmes in low- and middle-income countries: A systematic review and narrative synthesis. *Prev Med (Baltim)* 2021;143:106317.
18. Das JK, Salam RA, Lassi ZS, et al. Interventions for Adolescent Mental Health: An Overview of Systematic Reviews. *Journal of Adolescent Health* 2016;59(2):S49-S60.
19. Klasen H, Crombag AC. What works where? A systematic review of child and adolescent mental health interventions for low and middle income countries. *Soc Psychiatry Psychiatr Epidemiol* 2013;48(4):595-611.
20. Skeen S, Laurenzi CA, Gordon SL, et al. Adolescent mental health program components and behavior risk reduction: A Meta-analysis. *Pediatrics* 2019;144(2):e20183488.
21. Pedersen GA, Smallegange E, Coetzee A, et al. A Systematic Review of the Evidence for Family and Parenting Interventions in Low- and Middle-Income Countries: Child and Youth Mental Health Outcomes. *J Child Fam Stud* 2019;28(8):2036-2055.
22. Life in Lockdown: Child and adolescent mental health and well-being in the time of COVID-19. https://www.unicef-irc.org/publications/1227-life-in-lockdown.html. Accessed January 11, 2024.
23. The impact of COVID-19 on children’s mental health | UNICEF India. https://www.unicef.org/india/impact-covid-19-childrens-mental-health#. Accessed January 11, 2024.
24. UNICEF and WHO joint programme on mental health and psychosocial well-being and development of children and adolescents. https://www.who.int/publications/i/item/978924006176. Accessed January 11, 2024.
25. Novins DK, Stoddard J, Althoff RR, et al. Editors’ Note and Special Communication: Research Priorities in Child and Adolescent Mental Health Emerging From the COVID-19 Pandemic. *J Am Acad Child Adolesc Psychiatry* 2021;60(5):544-554.e8.
26. Price JC, Lee JJ, Saraiya N, et al. An Update on NIH Programs Relevant to Child Brain Health Research: ECHO, ABCD, HBCD, and MIRA. *J Neurosurg Anesthesiol* 2023;35(1):119-123.
27. COVID-19 Parental Resources Kit – Early Childhood. https://www.cdc.gov/mentalhealth/stress-coping/parental-resources/early-childhood/index.html. Accessed January 11, 2024.
28. COVID-19 Parental Resources Kit – Childhood. https://www.cdc.gov/mentalhealth/stress-coping/parental-resources/childhood/index.html. Accessed January 11, 2024.
29. COVID-19 Parental Resources Kit – Adolescence. https://www.cdc.gov/mentalhealth/stress-coping/parental-resources/adolescence/index.html. Accessed January 11, 2024.
30. CDC reports on teens’ mental health during pandemic reveal alarming trends | AAP News | American Academy of Pediatrics. https://publications.aap.org/aapnews/news/19913/CDC-reports-on-teens-mental-health-during-pandemic?autologincheck=redirected. Accessed January 11, 2024.
